# Supplementary material for: Pyruvate Dehydrogenase Kinase 1 inhibition mediated oxidative phosphorylation enhancement in cartilage promotes osteoarthritis progression
Source: BMC Musculoskelet Disord. 2023 Jul 20;24:597. doi: 10.1186/s12891-023-06585-6 (PMC10357736; doi:10.1186/s12891-023-06585-6)
Supplement: Supplementary file 1 — Additional file 1. [file 12891_2023_6585_MOESM1_ESM.zip › BP.Gsea.1653623667859/GOBP_OVULATION.html]

Details for gene set GOBP\_OVULATION[GSEA]

|  || Dataset | 55457\_collapsed\_to\_symbols.cls.cls #OA\_versus\_normal.cls.cls #OA\_versus\_normal\_repos |
| Phenotype | cls.cls#OA\_versus\_normal\_repos |
| Upregulated in class | normal |
| GeneSet | GOBP\_OVULATION |
| Enrichment Score (ES) | -0.56314117 |
| Normalized Enrichment Score (NES) | -1.7620542 |
| Nominal p-value | 0.0017699115 |
| FDR q-value | 0.114128344 |
| FWER p-Value | 0.998 |
Table: GSEA Results Summary

  

Fig 1: Enrichment plot: GOBP\_OVULATION      
 Profile of the Running ES Score & Positions of GeneSet Members on the Rank Ordered List

  

| SYMBOL | TITLE | RANK IN GENE LIST | RANK METRIC SCORE | RUNNING ES | CORE ENRICHMENT || 1 | HPGD | 15-hydroxyprostaglandin dehydrogenase [Source:HGNC Symbol;Acc:HGNC:5154] | 1941 | 0.368 | -0.1068 | No |
| 2 | GAS2 | growth arrest specific 2 [Source:HGNC Symbol;Acc:HGNC:4167] | 2866 | 0.264 | -0.1471 | No |
| 3 | AFP | alpha fetoprotein [Source:HGNC Symbol;Acc:HGNC:317] | 6561 | -0.047 | -0.4283 | No |
| 4 | TNFAIP6 | TNF alpha induced protein 6 [Source:HGNC Symbol;Acc:HGNC:11898] | 6799 | -0.063 | -0.4392 | No |
| 5 | MMP2 | matrix metallopeptidase 2 [Source:HGNC Symbol;Acc:HGNC:7166] | 7037 | -0.082 | -0.4478 | No |
| 6 | NRIP1 | nuclear receptor interacting protein 1 [Source:HGNC Symbol;Acc:HGNC:8001] | 8524 | -0.195 | -0.5398 | Yes |
| 7 | PLAT | "plasminogen activator, tissue type [Source:HGNC Symbol;Acc:HGNC:9051]" | 8689 | -0.208 | -0.5277 | Yes |
| 8 | LEP | leptin [Source:HGNC Symbol;Acc:HGNC:6553] | 8740 | -0.212 | -0.5063 | Yes |
| 9 | SIRT1 | sirtuin 1 [Source:HGNC Symbol;Acc:HGNC:14929] | 8947 | -0.227 | -0.4951 | Yes |
| 10 | PGR | progesterone receptor [Source:HGNC Symbol;Acc:HGNC:8910] | 9048 | -0.235 | -0.4748 | Yes |
| 11 | NOS3 | nitric oxide synthase 3 [Source:HGNC Symbol;Acc:HGNC:7876] | 9980 | -0.319 | -0.5091 | Yes |
| 12 | MMP19 | matrix metallopeptidase 19 [Source:HGNC Symbol;Acc:HGNC:7165] | 10652 | -0.388 | -0.5149 | Yes |
| 13 | EREG | epiregulin [Source:HGNC Symbol;Acc:HGNC:3443] | 10778 | -0.403 | -0.4765 | Yes |
| 14 | IL4R | interleukin 4 receptor [Source:HGNC Symbol;Acc:HGNC:6015] | 10902 | -0.417 | -0.4363 | Yes |
| 15 | INHBA | inhibin subunit beta A [Source:HGNC Symbol;Acc:HGNC:6066] | 11237 | -0.462 | -0.4072 | Yes |
| 16 | FOXO3 | forkhead box O3 [Source:HGNC Symbol;Acc:HGNC:3821] | 11305 | -0.472 | -0.3561 | Yes |
| 17 | RGS2 | regulator of G protein signaling 2 [Source:HGNC Symbol;Acc:HGNC:9998] | 12039 | -0.603 | -0.3411 | Yes |
| 18 | ADAMTS1 | ADAM metallopeptidase with thrombospondin type 1 motif 1 [Source:HGNC Symbol;Acc:HGNC:217] | 12553 | -0.775 | -0.2884 | Yes |
| 19 | PTGS2 | prostaglandin-endoperoxide synthase 2 [Source:HGNC Symbol;Acc:HGNC:9605] | 12641 | -0.820 | -0.1973 | Yes |
| 20 | INHBB | inhibin subunit beta B [Source:HGNC Symbol;Acc:HGNC:6067] | 12680 | -0.851 | -0.0987 | Yes |
| 21 | MYC | "MYC proto-oncogene, bHLH transcription factor [Source:HGNC Symbol;Acc:HGNC:7553]" | 12801 | -0.970 | 0.0077 | Yes |
Table: GSEA details [plain text format]

  

Fig 2: GOBP\_OVULATION      
 Blue-Pink O' Gram in the Space of the Analyzed GeneSet

  

Fig 3: GOBP\_OVULATION: Random ES distribution      
 Gene set null distribution of ES for **GOBP\_OVULATION**
